# A scoping review exploring the health impact and sustainability of collaborative paediatric heart disease treatment programmes in developing countries

**DOI:** 10.3389/fped.2026.1921366

**Published:** 2026-08-11

**Authors:** S. K. Sulaiman, N. Hamza, M. Martin-Khan

**Affiliations:** 1 Exeter Medical School, University of Exeter, Exeter, United Kingdom; 2 School of Nursing, University of Northern British Columbia, Prince George, BC, Canada

**Keywords:** congenital heart disease, global health, health impact, paediatric, pediatric, programmes, sustainability

## Abstract

**Introduction:**

The global burden of paediatric heart disease is increasing with widening disparities between developed countries and developing economies. In the last two decades, mortality and morbidity rates have markedly improved in developed nations, however they continue to rise in developing countries. Collaborative treatment programmes are crucial for bridging this gap where the necessary healthcare infrastructure is lacking. This scoping review aims to map the health impact, economic outcomes, and capacity building initiatives of collaborative paediatric heath disease treatment programmes in developing countries.

**Methods:**

A comprehensive search was conducted across MEDLINE, Embase and Global Health from inception to May 5th, 2024. Studies were screened by title and abstract, followed by full-text review, applying predefined inclusion and exclusion criteria. Data on patient and programme characteristics, mortality rates, economic impact and capacity-building initiatives were extracted and analysed.

**Results:**

The review included 35 studies. Thirty studies reported on mortality rates which ranged from 0% to 12.4%. Nine studies reported on mortality pre and post programme with a 4% to 41% reduction in mortality. Many studies included capacity building initiatives within their programmes. This included: Training and Education (*n* = 26); Infrastructure Development (*n* = 13); and Systems Strengthening (*n* = 18). Long-term programmes placed more than double the emphasis on systems strengthening approaches compared to short-term missions.

**Conclusion:**

Our review extensively maps the various characteristics and approaches of collaborative paediatric heart disease programmes. Overall, the findings suggests that these initiatives have the potential to provide favourable clinical outcomes and if setup appropriately they can support development of local expertise, improve healthcare infrastructure, and promote self-sustaining services in developing countries. A multi-faceted approach is required to improve paediatric cardiac care globally. Evidence evaluating long term impact and sustainability remains limited and further research is required to better understand how collaborative models can be optimised to support equitable and sustainable paediatric cardiac care globally.

## 1 Introduction

Paediatric heart disease presents a significant global challenge. This includes both congenital and acquired heart diseases however congenital heart disease (CHD) makes up majority of the cases. The prevalence of CHD globally is approximately 1.8% of all live births (1), contributing to one-third of all birth defects (1, 2). In developing economies, the prevalence is reportedly higher due to elevated birth rates (3), and the interplay between genetics and environmental factors (4). In developed countries the mortality from CHD has improved significantly allowing children with conditions once deemed untreatable to now survive into adulthood (5). However, in low and middle-income countries the morbidity and mortality are continuing to rise (6) resulting in a sustained global disparity in paediatric heart disease between developed and developing countries despite treatment advances (7).

Effective management of paediatric heart disease needs significant resources and a complex infrastructure, which is influenced by factors such as funding, healthcare expertise, and the overall health infrastructure (8). Humanitarian efforts by non-governmental organisations (NGOs), government, and other private stakeholders have a role in bridging the gap in many developing countries that lack the complex infrastructure needed to manage paediatric heart disease (9). The introduction of the Sustainable Development Goals (SDGs) 2030 agenda outlined an approach to establish a global framework to advance human development and promote sustainable development worldwide (10). A literature review by Ghandour identified that 15 SDGs of the 17 were impacted by cardiothoracic community, most significantly SDG 3, which focuses on health and well-being including targets relating to global maternal mortality ratio (target 3.1), preventable deaths of newborns and children (target 3.2), and achieving universal health coverage (target 3.8) (11). Consequently, failure to address the growing burden of congenital heart disease in developing countries may hinder progress towards achieving SDG 3 and potentially other interconnected Sustainable Development Goals (11).

Several programmes have reported on their individual outcomes. However, there is a lack of research comparing and evaluating the effectiveness of these efforts in the context of paediatric heart disease. Consequently, each organisation operates based on its own remits and judgement. Identifying which strategies are most effective is crucial for directing resources appropriately and informing policy and allocation of government resources. Therefore, we conducted this scoping review to map the health impact and sustainability of collaborative paediatric heart disease treatment programmes in developing countries.

## 2 Methods

This study utilised a scoping review methodology due to the heterogeneous nature of the research area, and the broad reach of the research question. A scoping review allowed us to capture the anticipated diverse nature of CHD programmes and the wide variation in the implementation and outcomes.

This review was conducted based on a pre-designed protocol following the Arksey and O'Malley framework for scoping reviews (2005) which included: (1) identification of the research question; (2) identification of relevant studies; (3) study selection; (4) data collection; and (5) analysis and reporting of results (12). The review was reported using the PRISMA-ScR (Preferred Reporting Items for Systematic reviews and Meta-Analyses extension for Scoping Reviews) guidelines (13) which also apply to scoping reviews.

### 2.1 Ethical considerations

Ethical approval was obtained by completing an ethical approval form with a review protocol and submitting to the University of Exeter. This review did not involve handling any patient sensitive data, however ethical considerations were addressed by ensuring proper citation of all sources and maintaining data integrity throughout. The methodology was transparently documented to support reproducibility and allow for independent verification of the review process.

### 2.2 Phase 1: identification of the research question

The PICO framework tool (Patient/population; Intervention; Comparison; Outcome) was used to formulate the research question and guide the search strategy (14).

The research question was:

“What is the health impact and sustainability of collaborative paediatric heart disease treatment programmes in developing countries?”

In this question, collaborative programmes included international, regional, or domestic partnerships involving two or more institutions working together to offer paediatric cardiac services.

We defined sustainability as programmes with local workforce development, progressive local independence, and integration into routine healthcare delivery. There were no direct comparators identified.

Our objectives were:

1. To demonstrate the health outcomes of collaborative treatment programmes.
2. To describe their economic outcomes.
3. To characterise the capacity-building efforts across different programmes.

### 2.3 Phase 2: identification of relevant studies

#### 2.3.1 Search strategy

The following databases were searched from inception up until May 5th, 2024: MEDLINE, Embase and Global Health via Ovid platform. The search strategy was developed using combination of Medical Subject Headings (MeSH) with search string developed for three main key concepts (congenital heart disease, paediatrics, and programme) (Table 1). The database search was limited to title and abstract to give highly relevant results. The electronic database search was supplemented by a Google search limited to the first 2,000 entries and sorted by relevance. The Google search was conducted using the same three key concept terms to identify additional publications.

**Table 1:** Search strategy of databases.

#	Key Concept	Search string
1	Congenital heart disease	Congenital heart disease.ti. OR (MM “Heart Defects, Congenital+”) OR (congenital heart defect or congenital heart diseases or defect OR congenital heart or disease OR congenital heart or heart defect OR congenital or heart defects OR congenital or heart disease, congenital).tw.
		OR congenital cardiac.tw.
		OR (MM “Cardiac Surgical Procedures+”)
2	Paediatrics	Pediatric.tw or Pediatrics OR Paediatric*.tw OR child.tw or (MM “Adolescent”) OR (MM “Child+”) OR (MM “Preschool”) OR (MM “Infant”)
3	Programme	Program$1.tw OR Programme*1.tw OR Initiative$.tw OR Project$.tw OR Campaign$.tw OR Service$.tw OR (MM “Health Promotion+”) OR collaborative.tw OR early medical intervention.mp or (MM “Early Medical Intervention”)
4	1 AND 2 AND 3	
5	Filter	Search in Title and Abstract only

MM, MeSH heading; *.mp*, multi-word phrase;*.ti*, title/abstract;.*tw*, text word.

#### 2.3.2 Selection criteria

The inclusion and exclusion criteria were established in the protocol at the start of the review (Table 2). All peer reviewed literature of variable study designs (e.g., randomised controlled, cohort, case study, observational, cross-sectional studies) published in English up until 5th May 2024, were included in the review. Published conference abstracts, editorials or commentaries were excluded. Grey literature was also excluded.

**Table 2:** Study inclusion and exclusion criteria used for article selection.

Category	Inclusion criteria	Exclusion criteria
Patient/Population	Studies where the population of interest was children or young people (0–18y)	Studies with children and adults
Diagnosis	Diagnosed with paediatric heart disease (congenital/congenital and acquired)	Studies only focussing on acquired paediatric heart disease
Population	Programmes operating in developing economies, as classified by the United Nations (59).	Exclusively based in economies classified as “economies in transition” or developed countries.
Intervention	Treatment programme	Programmes limited to prevention or screening without therapeutic components.
		Established as a registry solely for data collection
Stakeholders	Collaborative programmes involving two or more stakeholders.	Standalone programmes (60–67)
Setting	Programmes situated in diverse healthcare settings such as hospitals, outpatient clinics, and community health centres.	

### 2.4 Phase 3: study selection

#### 2.4.1 Data screening

The database search was initially conducted by one author [SKS] who uploaded the search results into a review software called Covidence (15). Duplicates were removed and screening done within Covidence. The same author screened the titles and abstracts using Covidence and continued with full-text screening of the articles. Screening was done against the designed protocol following discussion between all authors. To reduce selection bias, two reviewers [MMK and NH] reviewed a sample of the identified articles which showed consistency in selection. Further, uncertain articles were also discussed with two reviewers [MMK and NH] who did additional full-text screening of the disputed articles, and any discrepancies were resolved by discussion until the reviewers reached a conclusion. The final list of included studies was reviewed and agreed by all authors.

### 2.5 Phase 4: data collection

#### 2.5.1 Data extraction and mapping

Data extraction was performed using a data extraction form template developed in Covidence (15). The form included publication information (title, publication year and journal), study information (aim, study design, country and city and timeframe of study), method, programme description (geographical location, inclusion and exclusion criteria, referral pathway, scope, logistics, intervention), patient description (sample size, age, weight), and results (health outcomes, economic outcomes, and capacity building outcomes).

The data was exported to Microsoft Excel [Version 16.95.1] and mapped in a descriptive table (Table 4). For ease of interpretation this was further grouped into separate summary tables looking at health outcomes (Supplementary Table 1), economic outcomes (Table 4), and capacity building initiatives (Table 6).

#### 2.5.2 Critical appraisal

Consistent with scoping review methodology, a critical appraisal and risk-of-bias assessment of the included studies was not undertaken.

### 2.6 Phase 5: analysis and reporting of results

#### 2.6.1 Analysis

We grouped programmes based on different characteristics. The included studies were then separately evaluated against the three study objectives. Health outcomes were summarised using clinical indicators (mortality, post operative complications and hospital stay). Mortalities data were extracted as reported by individual studies and summarised descriptively. Capacity building initiatives were reported via measures of training, workforce development, and local service autonomy. The categories were grouped into three broad domains (Table 3) based on the components with WHO Health Systems Framework (16). Findings across these domains were then analysed against various programme characteristics to describe the overall sustainable impact of various programmes. Economic impact was demonstrated through available cost and resource data.

**Table 3:** Codebook used to classify capacity building measures identified in the included studies.

Category	Definition	Examples
Training and Education	Training and educational initiatives aimed at improving knowledge and clinical skills of the local workforce	Surgical mentoring, ICU teaching, simulation, workshops, fellowships
Infrastructure Development	Development and provision of physical resources and equipment	Operating theatres, echocardiography machines, ICU equipment, catheter laboratory development
Systems Strengthening Approaches	Activities supporting clinical governance, organisation, data systems, quality improvement and service integration	MDT establishment, referral pathways, databases, governance, telemedicine, quality improvement

No meta-analysis or pooled quantitative synthesis was undertaken because of heterogeneity in study design, programme characteristics, patient populations, and outcome reporting.

## 3 Results

### 3.1 Description of the studies

**Figure 1:**
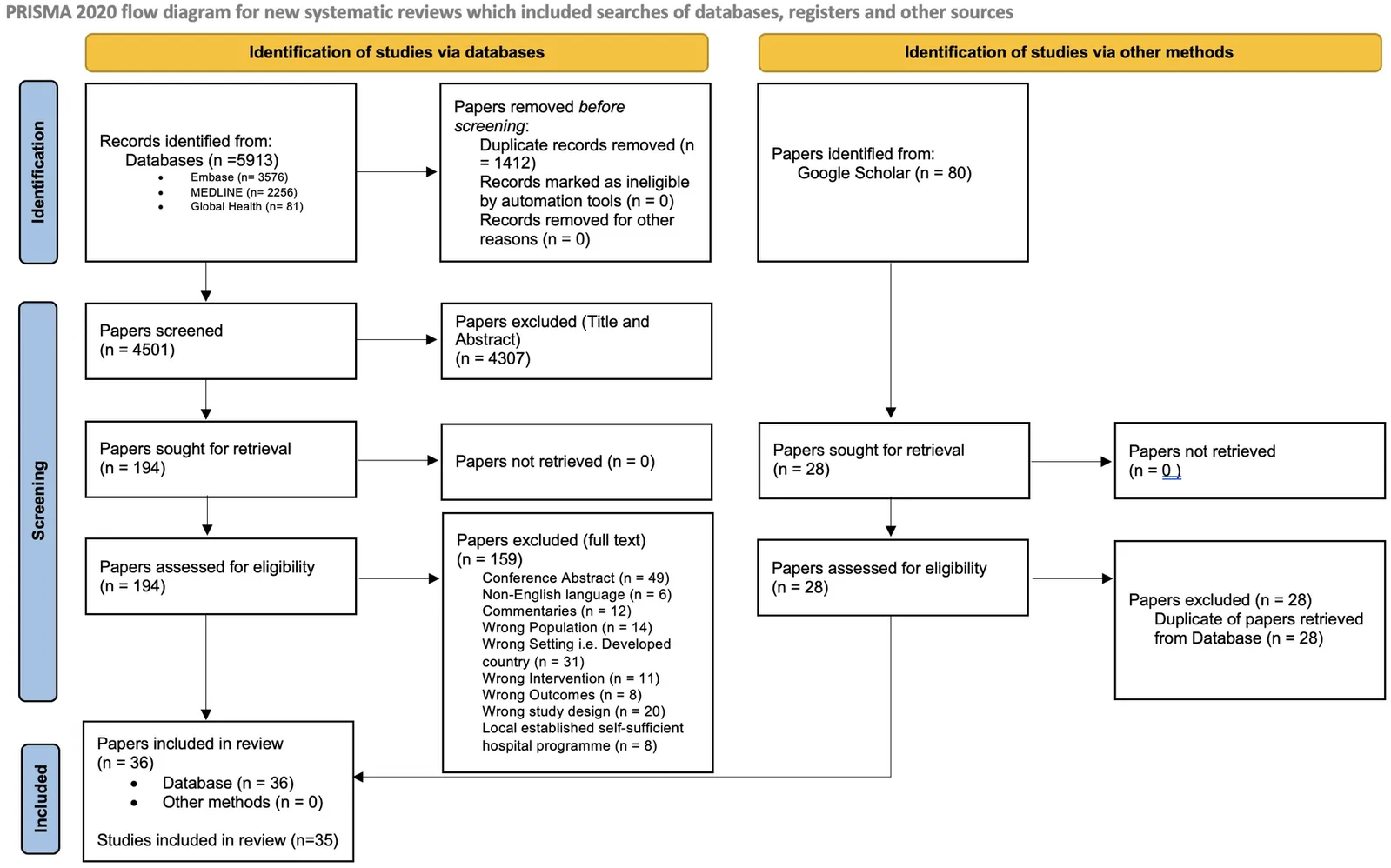
PRISMA flow diagram illustrating the study identification, screening, eligibility assessment, and inclusion process.

There were 5,914 records identified from databases and 80 records from a supplementary search on Google Scholar. After the removal of duplicate records, 4,501 records remained for title and abstract screening. From these records 222 papers were sought (194 from database and 28 from Google scholar) and assessed for eligibility by full text review. The papers from Google Scholar were duplicates of those already obtained from the databases search hence all 28 papers retrieved from Google Scholar were excluded. Following full text screening, 36 papers were included in the review. One study was published twice in different papers (17, 18) so this was included as one study for the rest of the review (18). Hence a total of 35 studies from 36 papers were included for further analysis (Table 4). The search and screening stages, including the reasons for exclusion, are outlined in the PRISMA-ScR flow diagram (Figure 1).

**Table 4:** Overview of all included studies with associated patient and programme characteristics.

Study ID	Participating country	Start date of study	End date of study	Sample size	Number of procedures	Females (%)	Type of paediatric heart disease	Partnership model	Scope	Logistics	Duration of stay
Ali & Medani (19)	Sudan	2004	2011	–	–	–	Congenital and Acquired	2P	National	On-site and Fly-in	Long term
Balachandran et al. (33)	India	Jan-10	Dec-12	1,702	–	45%	Congenital	2P	International	On-site	Long term
Bawiskar et al. (34)	India	2018	2019	130	115	–	Congenital	2P	National	On-site	Long term
Cardarelli et al. (22)	Global	Jan-15	Dec-15	446	446	43%	Congenital	2P	International	Fly-in	Short term missions
Cohen et al. (52)	Global	1995	2000	386	386	–	Congenital	2P	International	Fly-in and Fly-out	Hybrid
Croti et al. (28)	Brazil	Jan-11	Dec-17	1,202	1,202	–	Congenital and Acquired	3P	International	On-site	Long term
Das et al. (35)	India	Aug-13	Dec-22	27,844	22,572	–	Congenital	3P	National	On-site	Long term
Rassi et al. (50)	Lebanon	Jan-14	Dec-18	856	993	43%	Congenital	3P	Local	On-site	Long term
Furniss et al. (23)	Global	Jan-08	Dec-17	100	97	47%	Congenital	2P	International	Fly-out	Short term missions
Henry et al. (32)	Trinidad and Tobago	Sep-98	Feb-03	204	208	–	Congenital	2P	National	On-site and Fly-in	Long term
Ho et al. (38)	China	Nov-07	Jul-10	36	37	53%	Congenital	2P	Local	On-site	Long term
Isaac et al. (29)	Guyana	Feb-14	Jul-16	319	114	–	Congenital	2P	National	Fly-in	Short term missions
Kim et al. (37)	India	1988	2021	357	357	–	Congenital	3P	Local	On-site	Long term
Lacour-Gayet et al. (24)	Global	Jan-96	Jan-19	531	531	–	Congenital	2P	International	Fly-out	Hybrid
Larrazabal et al. (30)	Guatemala	Feb-97	Jul-04	1,215	1,215	58%	Congenital	2P	National	On-site	Long term
Leon-Wyss et al. (31)	Guatemala	Feb-97	Dec-07	2,630	2,630	–	Congenital	2P	National	On-site	Long term
Liu (39)	China	1984	2007	–	–	–	Congenital	3P	International	On-site and Fly-in	Long term
Marianeschi et al. (43)	Cambodia	2012	2019	114	114	–	Congenital	2P	International	Fly-in	Short term missions
Marianeschi et al. (25)	Global	2005	2020	–	–	–	Congenital and Acquired	2P	International	Fly-in	Short term missions
Nair et al. (36)	India	Aug-17	Aug-19	5,951	2,059	–	Congenital	2P	Local	On-site	Long term
Novick et al. (26)	Global	Apr-93	Mar-03	–	1,580	–	Congenital and Acquired	2P	International	Fly-in	Short term missions
Nunes et al. (47)	Angola	Apr-11	Aug-15	1,682	1,766	52%	Congenital and Acquired	3P	National	On-site	Long term
Park et al. (20)	Ethiopia and Cote d'Ivoire	2015	2019	–		–	Congenital and Acquired	2P	National	On-site and Fly-in	Long term
Piros Kulandasamy Pillai et al. (44)	Malayisa	Aug-08	Sep-12	230	230	46.10%	Congenital	3P	National	Fly-out	Short term missions
Sasson & Schachner (53)	Global	2013	2018	–	–	–	Congenital	2P	International	Fly-in	Hybrid
Shidhika et al. (48)	Namibia	2009	2015	193	156	56%	Congenital and Acquired	2P	National	On-site	Long term
Song et al. (40)	China and Cambodia	Jan-17	Jan-20	3,310	–	56.40%	Congenital	3P	National	On-site	Long term
Tefera et al. (49)	Ethiopia	Jan-09	Dec-12	287	296	65.20%	Congenital	2P	International	Fly-in	Short term missions
Tefuarani et al. (18)	Papua New Guinea	1978	1994	165	133	50%	Congenital	3P	National	Fly-out	Short term missions
Tefuarani et al. (45)	Papua New Guinea	1994	2006	470	470	56%	Congenital	3P	National	Fly-in	Short term missions
Tomita et al. (41)	Mongolia	2001	2011	–	314	–	Congenital	2P	National	Fly-in	Short term missions
Wallen et al. (27)	Global	2008	2016	3,783	3,783	48.70%	Congenital	2P	International	Fly-in	Short term missions
Youssef et al. (51)	Iraq	Oct-21	Oct-22	144	148	49%	Congenital	2P	National	Fly-in	Long term
Zain et al. (46)	Malaysia	Sep-15	Nov-18	60	60	–	Congenital	2P	Local	Fly-in	Short term missions
Zhang et al. (42)	China	2014	2019	240	226	–	Congenital	2P	Local	Fly-in	Long term

### 3.2 Characteristics of included studies

Thirty-five studies (from 36 papers) were included in the review with a publication range of 23 years (2001–2024). Thirty-three of the studies included were observational, cohort studies with a quantitative methodology. The remaining two studies were both mixed methods; one study was descriptive (19); and one was a cohort study (20).

**Figure 2:**
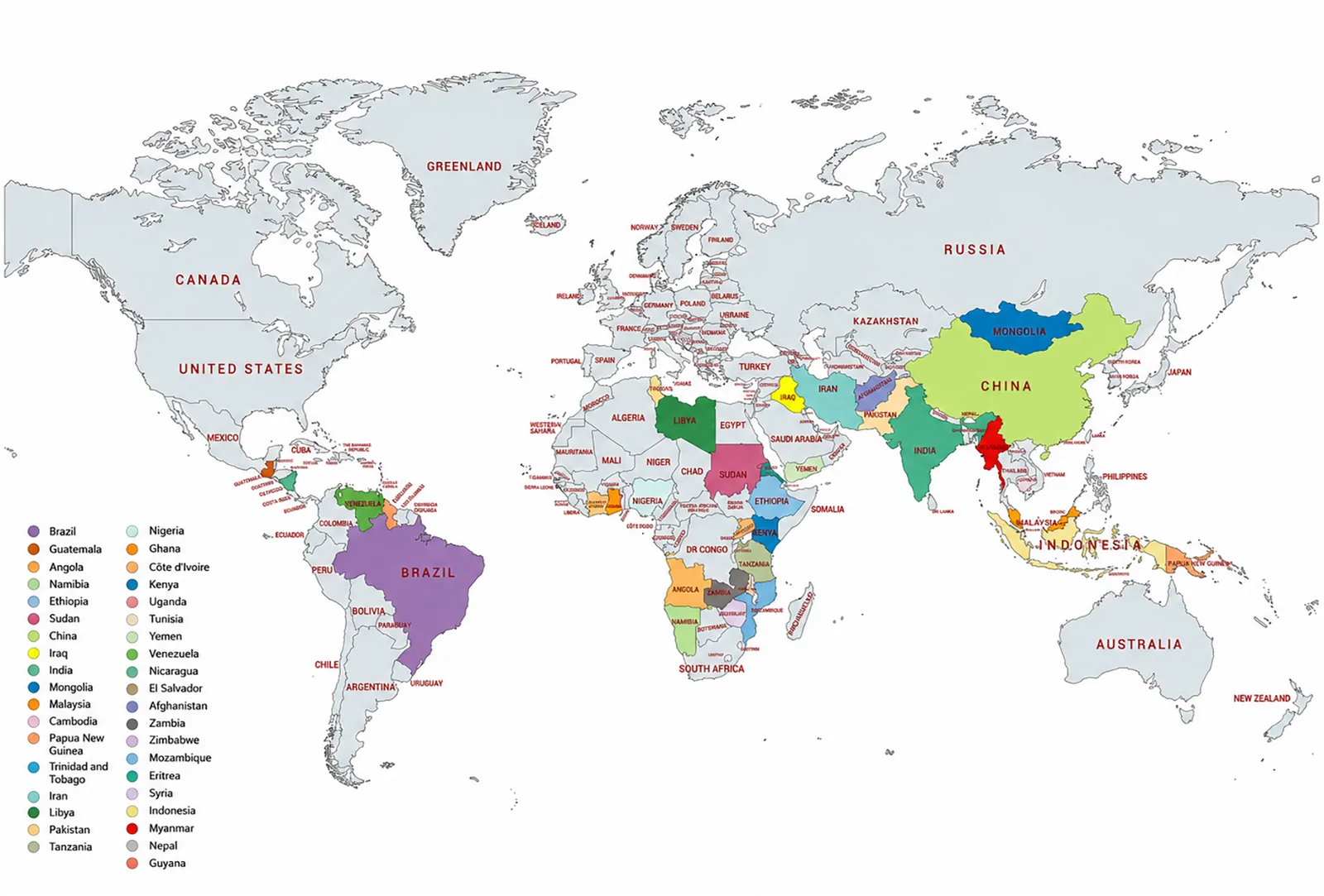
Map showing geographic distribution of collaborative paediatric heart disease treatment programmes included in the review.

There were 32 participating countries included with good geographical diversity (Figure 2). These were grouped further into regions based on the classification by United Nations (21). There were six studies that were global (22–27), two conducted in South America (28, 29), two in Central America (30, 31), one in Caribbean (32), five in Southern Asia (33–37), five in South-eastern Asia (38–42), five in Eastern Asia (18, 43–46), five in Africa (19, 20, 47–49), two in Western Asia (50, 51).

The sample size was reported in 28 studies (18, 22–24, 27–38, 40, 42–52). The total number of participants (*n* = 28/35 studies; 80%) was 54,587 patients (Mean = 1,950; Median = 372 patients; Range = 60–27,844). The total number of procedures reported (*n* = 28/35 studies) (18, 22–24, 26–32, 34–38, 42–52) was 42,238 (Mean: 1,509; Median: 226; Range 60–22,572).

Demographics were poorly reported with inconsistencies between studies. Gender was reported in less than half the studies (*n* = 15/35; 43%) (18, 22, 23, 27, 30, 33, 38, 40, 44, 45, 47–51). On average, males and females were evenly distributed (Females, Mean: 51%). The median age (reported in 12 studies) averaged 4.1 years (18, 23, 24, 27, 30, 33, 35, 38, 40, 43, 45, 46). The average age (*n* = 7/35; 20%) was 5.8 years (18, 22, 27, 29, 38, 49, 50).

All programmes provided secondary and tertiary level of care to patients. Secondary care was defined as specialist care at the first referral level by a specialist. Tertiary level care referred to highly advanced specialised care delivered for complex conditions. There were a range of different programme characteristics. Below, key characteristics are reported, with a detailed summary provided in Table 4.

#### 3.2.1 Partnership types

**Figure 3:**
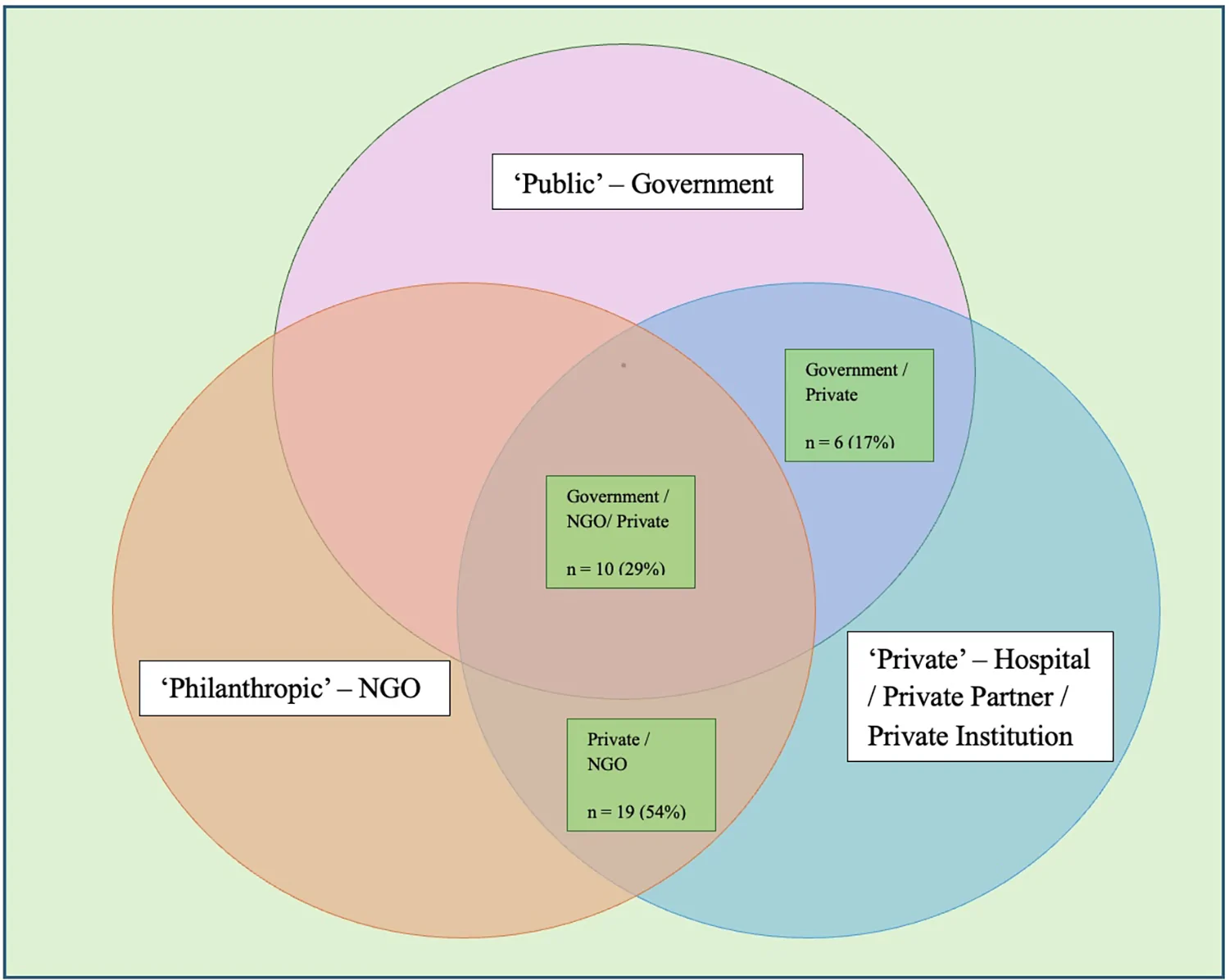
Distribution and overlap of 2P and 3P stakeholder models for collaborative paediatric heart disease treatment programmes.

After reviewing all the studies three key stakeholders were identified for partnership programmes. These were public organisations which were usually government based, philanthropic which were often described as NGO, and private, which included, private hospital, private organisation, private partner. These stakeholders were grouped in either Two-Partner (2P) Models and Three-Partner (3P) Models (Figure 3).

There was a total of 25 studies with Two-Partner Models (2P). This was the most common partnership model. Of the 25, six studies (32–34, 36, 48, 51) were collaborations between government entities and private sector organisations (public-private partnership) and 19 studies (19, 20, 22–27, 29–31, 38, 41–43, 46, 49, 52, 53) were between private companies and NGOs (Private-NGO partnership).

The Three-Partner Models (3P) were more complex models incorporating public, private, and NGO partnerships working together in a coordinated effort. There were a total of 10 studies with the 3P model (18, 28, 35, 37, 39, 40, 44, 45, 47, 50).

#### 3.2.2 Scope of programmes

The geographical scope of the programmes varied as some initiatives focused solely on treating patients in specific local areas, whilst others provided care to patients across the entire country, and some even extended their services internationally. The most common was for programmes to provide a national scope (*n* = 16) (18–20, 29–32, 34, 35, 40, 41, 44, 45, 47, 48, 51). These national programmes employed both partnership models with 56% using the 2P model (19, 20, 29–31, 34, 41, 48, 51) and 44% using the 3P model (18, 32, 35, 40, 44, 45, 47).

There were 13 studies that targeted participants across multiple countries or regions (22–28, 33, 39, 43, 49, 52, 53). These international initiatives often operated in 2P model partnerships with one international programme operating as a 3P model (39).

Few studies had programmes that operated with a specific design to serve only local communities (*n* = 6/35) (36–38, 42, 46, 50). These local programmes were often highly tailored to meet the specific healthcare needs of the population they served, utilising local resources and using a 2P partnership that allowed for more targeted interventions.

#### 3.2.3 Logistics

The logistics of implementation of the programmes differed across the studies, influencing how care was delivered and accessed by the target populations. We identified four major models for delivering the programme, which included on-site, fly-in, fly-out, and combination models.

Thirteen programmes were conducted “on-site”, meaning that patients received treatment within their own country and local area (28, 30, 31, 33–38, 40, 47, 48, 50). Another model was the “fly-in” approach, observed in 13 studies (22, 25–27, 29, 41–43, 45, 46, 49, 51, 53). In this model, medical teams were flown into the country where the patients resided to provide care. A “fly-out” model, where patients were transported to another country for treatment, was only documented in four studies and was utilised when the required medical expertise or facilities were unavailable locally (18, 23, 24, 44). Additionally, there were five studies that employed a combination of these approaches, leveraging the strengths of both models (19, 20, 32, 39, 52).

#### 3.2.4 Duration of stay of programmes

In examining the duration of stay, we focused on how long each programme stayed on the ground as this provides insight into the nature and depth of engagement. The programmes were categorised into short term missions, long term programmes, and hybrid models.

Short term missions were interventions that lasted a few days to few weeks at a time. These missions were typically conducted on a recurring basis, either annually or bi-annually. A total of 13 programmes were identified as short-term missions (18, 22, 23, 25–27, 29, 41, 43–46, 49). Long term programmes, were characterised by their sustained presence, extending continuously over a period of more than one year. A total of 19 studies had long term programmes (19, 20, 28, 30–40, 42, 47, 48, 50, 51). Lastly, hybrid models combined elements of both short-term and long-term approaches. Only three studies utilised this combination of short-term and long-term strategies (24, 52, 53).

### 3.3 Health outcomes

The health outcomes were characterised by a combination of measures across the various studies, including mortality (30 studies), complications (15 studies) and Length of Stay (LOS) (9 studies) and Intensive Care Unit (ICU) stay (9 studies). An overview is provided here, and more details are in the Supplementary Table 1.

#### 3.3.1 Mortality

Mortality reporting was heterogenous, and studies reported mortality at varying intervals and using various definitions including in-hospital, peri or post op mortality, 30-day, 1 year, 5 year, or overall mortality with no specified follow up period, thus limiting comparability between programmes. Across included studies, the reported mortality rates ranged from 0% to 12.4%.

Eight studies reported in-hospital mortality, 5 reported peri or post op mortality, 4 reported 30-day mortality, 1 reported 1-year mortality, 1 reported 5-year mortality, and 11 reported overall mortality with no timing specified of period of follow up (see Supplementary Table 1).

Some studies (9/35) also measured percentage of mortality reduction over the study duration (26–29, 31, 33, 36, 37, 39). This ranged from 4% to a 41% reduction in mortality across the studies where this was mentioned.

Few studies (5/35) reported on Risk Adjustment for Congenital Heart Surgery (RACHS-1) category-wise mortality rate (27, 35, 37, 47, 50). In most of these studies (4/5) there was a clear trend of increasing mortality rate with higher RACHS categories reflecting the complexity and risk associated with these surgeries. However, one study reported a lower mortality rate for RACHS categories 5 and 6 (3.5%) compared to RACHS Category 4 (7.4%) (50). In the higher RACHS categories 5 and 6, mortality rates were notably high, with Das (2024) reporting a rate of 45.4% (35) and Nunes (2017) reporting 25% (47), while the lowest reported rate in this category was 3.5% (50).

The Standardised Mortality Ratio (SMR) compares observed deaths in a study population to expected deaths based on a reference population with paediatric heart disease. Two studies reported SMR data (28, 30) with both showing a reduction in the duration the study. One study reported a reduction in SMR from 2.64 in 2011 to 1.10 in 2017 (28). Another study showed a decrease in SMR from 10.0 (1997–1999) to 5.7 (2003–2004), with a statistically significant reduction compared to the earlier period (*p* = 0.008) (30).

#### 3.3.2 Complications

Fifteen studies reported on short term complications following a cardiac intervention. Infection was the most reported complication and mentioned in nine studies (28, 30–33, 38, 47, 48, 50). Bleeding was reported in five studies (27, 30–32, 48). Pulmonary complications like for instance pneumonia, pleural effusion, chylothorax or pneumothorax was noted in eight studies (8/15) (18, 23, 30, 31, 42, 47, 48, 50). Cardiac complications including arrhythmias were reported in seven studies (18, 23, 30–32, 47, 48). Re-operation due to acute surgical complications such as bleeding or residual defects was reported in seven studies (27, 32, 42, 45, 47, 49, 50).

Long term complications were not well reported or acknowledged in many studies. Three studies reported on pacemaker insertion due to heart block (23, 32, 47). One study reported stroke resulted in long term neurological deficit (49) and one study reported new-onset epilepsy following surgery (42).

#### 3.3.3 Length of stay (LOS) in hospital

The median or mean LOS was reported in eight studies (23–25, 30, 31, 47, 50, 51). The mean length of stay in the studies where this was reported ranged from 5 to 12.2 days (24, 25, 50, 51) and median length of stay was 6–12 days in those studies (23, 30, 31, 47).

#### 3.3.4 Intensive care unit (ICU) stay

The ICU stay was reported in studies (9/35) (23, 25, 30, 31, 33, 47, 48, 50, 51). The mean length of ICU stay ranged from 3 to 5.7 days (25, 50, 51) and median length of ICU stay ranged from 1 to 5 days (23, 30, 31, 33, 47, 48).

### 3.4 Economic impact

Economic impact data was inconsistently reported across the studies reviewed. Only six studies addressed economic outcomes and costs with delivering the programme and these varied significantly (Table 5). The cost per surgery was detailed in just three studies, with figures ranging from $866 to $2,400 and there was no specified breakdown of how the cost of surgery was calculated. Costs were not pooled or compared because of heterogeneity in the years, programme scope, costing methods, and healthcare settings.

**Table 5:** Overview of reported economic outcomes of collaborative paediatric heart disease treatment programmes.

Study	Year	Location	Total cost of programme ($ USD)	Cost per surgery ($ USD)	DALYs (Total life years gained)
Cardarelli et al. (50)	2018	Global	$3,210,873	–	15,561
Rassi et al. (50)	2020	Lebanon	$16,948,800	–	–
Kim et al. (37)	2023	India	–	$2,400	–
Marianeschi et al. (43)	2021	Cambodia	$190,581	$1,534	5,364
Marianeschi et al. (25)	2022	Global	–	$866	–
Novick et al. (26)	2005	Global	$4,452,264	–	–

The Disability-Adjusted Life Years (DALYs) averted was mentioned in two studies, ranging from 5,364 to 15,561 life years.

### 3.5 Capacity building initiatives

The capacity-building efforts of the programmes were independently explored across three key areas: Training and Education, Infrastructure Development, and Systems Strengthening Approaches (Table 6). Notably, nine studies incorporated all three capacity-building initiatives (19, 20, 25, 29–31, 39, 43, 53).

**Table 6:** Summary of reported capacity building initiative and health outcomes in collaborative paediatric heart disease treatment programmes.

Study ID	Scope	Duration of programme	Logistics	Training and education	Infrastructure development	Systems strengthening approaches	Mortality (%)	Mortality reduction (%)
Ali & Medani (19)	National (2P)	Long	On-site and Fly-in	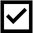	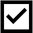	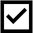	–	–
Balachandran et al. (33)	International (2P)	Long	On-site	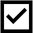	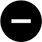	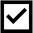	3.10%	2.10%
Bawiskar et al. (34)	National (2P)	Long	On-site	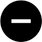	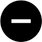	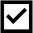	–	–
Cardarelli et al. (22)	International (2P)	Short	Fly-in	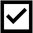	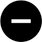	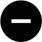	8.00%	–
Cohen et al. (52)	International (2P)	Hybrid	Fly-in and Fly-out	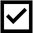	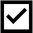	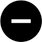	5.2%	–
Croti et al. (28)	International (2P)	Long	On-site	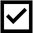	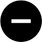	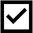	12.40%	7.00%
Das et al. (35)	National (3P)	Long	On-site	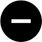	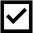	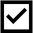	5.20%	–
Rassi et al. (50)	Local (3P)	Long	On-site	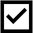	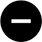	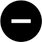	2.80%	–
Furniss et al. (23, 32)	International (2P)	Short	Fly-out	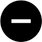	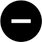	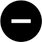	2.9%	–
Henry et al. (32)	National (2P)	Long	On-site and Fly-in	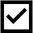	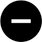	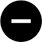	3.40%	–
Ho et al. (38)	Local (2P)	Long	On-site	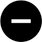	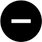	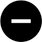	5.40%	–
Isaac et al. (29)	National (2P)	Short	Fly-in	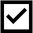	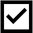	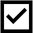	5%	4%
Kim et al. (37)	Local (3P)	Long	On-site	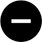	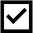	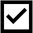	2.73%	8.00%
Lacour-gayet et al. (24)	International (2P)	Hybrid	Fly-out	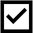	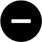	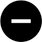	5.50%	–
Larrazabal et al. (30)	National (2P)	Long	On-site	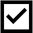	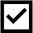	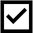	10%	–
Leon-Wyss et al. (31)	National (2P)	Long	On-site	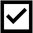	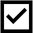	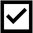	8.30%	2.00%
Liu (39)	International (3P)	Long	On-site and Fly-in	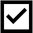	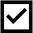	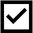	3%	7%
Marianeschi et al. (43)	International (2P)	Short	Fly-in	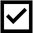	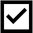	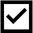	0.80%	–
Marianeschi et al. (25)	International (2P)	Short	Fly-in	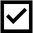	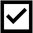	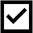	2%	–
Nair et al. (36)	Local (2P)	Long	On-site	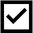	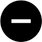	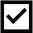	2.40%	41.00%
Novick et al. (26)	International (2P)	Short	Fly-in	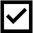	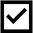	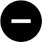	9.50%	8.70%
Nunes et al. (47)	National (3P)	Long	On-site	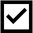	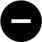	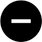	4.20%	–
Park et al. (20)	National (2P)	Long	On-site and Fly-in	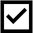	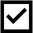	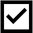	–	–
Piros Kulandasamy Pillai et al. (44)	National (3P)	Short	Fly-out	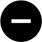	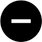	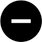	1.70%	–
Sasson & Schachner (53)	International (2P)	Hybrid	Fly-in	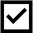	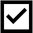	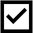	–	–
Shidhika et al. (48)	National (2P)	Long	On-site	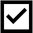	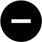	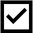	3.80%	–
Song et al. (40)	National (3P)	Long	On-site	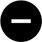	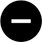	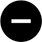	0.10%	–
Tefera et al. (49)	International (2P)	Short	Fly-in	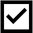	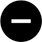	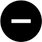	1%	
Tefuarani et al. (18)	National (3P)	Short	Fly-out	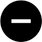	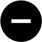	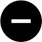	6%	–
Tefuarani et al. (45)	National (3P)	Short	Fly-in	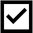	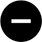	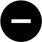	1.90%	–
Tomita et al. (41)	2P (National)	Short	Fly-in	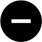	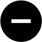	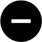	–	–
Wallen et al. (27)	2P (International)	Short	Fly-in	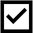	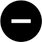	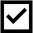	8.20%	5.80%
Youssef et al. (46, 51)	2P (National)	Long	Fly-in	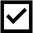	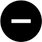	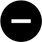	4.10%	–
Zain et al. (46)	2P (Local)	Short	Fly-in	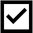	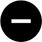	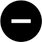	11%	–
Zhang et al. (42)	2P (Local)	Long	Fly-in	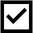	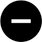	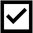	0%	–

#### 3.5.1 Training and education

Training and education were a focus in 26 studies with an aim to increase local autonomy and capacity (19, 20, 22, 24–33, 36, 39, 42, 43, 45–53). For example, Ali & Medani (19), Rassi et al. (50) and Lacour-Gayet et al. (24) discussed the value of fellowship programmes that facilitated exchanges between centres in developed and developing countries, enhancing the sustainability of paediatric cardiology programmes.

Whilst Larrazabal et al. (30) and Leon-Wyss et al. (31) discussed training clinicians locally by flying in the expertise into the centre. Liu (39) described a tailored approach whereby comprehensive team training was offered for units with regional leadership potential and medical mission support for less developed units, helping them manage complex cases.

#### 3.5.2 Infrastructure development

Infrastructure development was included in 13 programmes and focussed on various key areas (19, 20, 25, 26, 29–31, 35, 37, 39, 43, 52, 53). One major aspect was upgrading physical facilities for paediatric heart disease treatment through the renovation and expansion of hospitals, community centres, and surgical sites. For instance, Sasson and Schachner (53) highlighted that their programme was involved in establishing five independent cardiac surgical and interventional centres across countries (53). Similarly, Liu (39) highlighted how their programme improved infrastructure by setting up paediatric cardiology centres. Cohen et al. (52) in their programme helped in development of clinics which helped to upgrade necessary infrastructure.

Additionally, some studies concentrated on creating Paediatric Intensive care Units (PICU) and advancing medical equipment and technology. Larrazabal et al. (30) and Leon-Wyss et al. (31), for example, demonstrated how the creation of a new PICU improved post-operative management and reduced mortality. Other studies focused on establishing reliable supply chains for essential supplies and medications, as detailed by Park et al. (20) and Novick et al. (26).

#### 3.5.3 Systems strengthening approaches

Systems strengthening approaches, reported in 18 studies, involved policy implementation to enhance decision-making and healthcare management (19, 20, 25, 27–31, 33–37, 39, 42, 43, 48, 53).

For example, Croti et al. (28) described introducing a surgical safety checklist and new protocols developed aimed at improving patient safety, communication, post-operative care, and infection prevention (28). Another example is illustrated by Isaac et al. (29), which details the establishment of the Guyana Paediatric Cardiology Steering Committee. This committee improved patient triage and timely intervention, leading to better health outcomes.

### 3.6 Comparison between programme duration and outcomes

One of the major differences in programme characteristics was short term missions vs. long term programmes.

In short term missions, the mortality ranged from 0.8%–11%. Three of 13 programmes measured mortality reduction which ranged from 4%–8.7% (26, 27, 29). With regards to capacity building initiatives, 69% of short-term missions focussed on training and education (22, 25–27, 29, 43, 45, 46, 49) and 31% on infrastructure development (25, 26, 29, 43). Only 31% of short-term missions focussed on systems strengthening approaches (25, 27, 29, 43).

In long term programmes, the mortality ranged from 0%–10%. Six of 19 programmes measured mortality reduction which ranged from 2%–41% (28, 31, 33, 36, 37, 39). With capacity-building initiatives, 74% of long-term programmes focussed on training and education (19, 20, 28, 30–33, 36, 39, 42, 47, 48, 50, 51), and 37% focussed on infrastructure development (19, 20, 30, 31, 35, 37, 39). Systems strengthening approaches were included in 68% of long-term programmes (19, 20, 28, 30, 31, 33–37, 39, 42, 48).

## 4 Discussion

Organised and collaborative efforts are necessary in reducing the global disparities and improving the management of paediatric heart disease in children. This scoping review summarises the various collaborative paediatric heart disease programmes operating in developing countries and gives a picture into the health impact and sustainability of the programmes. To our knowledge, a scoping review of this kind has never been done before on this subject.

All the programmes in our review operated within either a 2P or 3P model. The 2P model was more commonly used than the 3P model, likely due to the comfort level of the programmes in forming collaborations. However, collaboration remains a challenge for paediatric cardiac programmes, and part of this is likely because they are often small (54) and struggle to secure enough support for 3P partnerships. This review also revealed that NGOs were involved in more programmes than governments. Government involvement is crucial for securing public funding and developing necessary infrastructure hence the lack of this can be a limitation in the success of these programmes (55). Kim (37) and Das (35) also highlighted that Public-Private models, or “twinning partnerships” where the government is involved improve the sustainability of programmes. NGOs often face funding challenges that limit their ability to scale up, and government support could alleviate this issue (54).

Onsite and fly-in programme logistics were the most utilised, with only four studies describing a fly-out programmes. Fly-out programmes tend to be more expensive and difficult to sustain long-term. For example, Novick (26) compared the cost per child between different years of their program. In the initial years, children were flown to the United States for surgery, while later, a fly-in approach was adopted. They found that the total cost per child for the children who received surgery in the United States was ten times higher than the cost per child in the overseas program. In later years, the fly-in approach was taken and proved to be more cost-effective and sustainable (26).

Regarding the duration of stay, short term missions, long-term, and hybrid programmes all have their individual roles in programmes. In this review, long-term programmes were more likely to include system-strengthening strategies than short-term missions. This is not surprising, as programmes that stay longer on the ground have more opportunities to influence strategic and policy changes and provide direction to local units.

Our review of the health outcomes found that mortality was the commonly studied health outcome however morbidity, particularly long term complications were not widely discussed. This could be due to the lack of follow up data due to constraints of many programmes.

The average mortality from Society of Thoracic Surgeons (STS) and European Association for Cardio-Thoracic Surgery (EACTS) is quoted at 3.8% (3.5% STS, 4.2% EACTS) (56). Of 30 studies that reported on mortality, 15 studies had a mortality rate within the average quoted by international cardiac associations. However, these results should be compared very cautiously due to differences in pathology, surgical complexity, patient selection, and follow-up durations which limit comparability with the scale of work in established congenital cardiac centres in developed countries.

Mortality can vary widely based on various patient factors including the complexity of the surgery. RACHS scoring system is a useful way of quantifying complexity of surgery. However, only 5 studies within this review showed RACHS-1 categories of patients and associated mortality rates. A higher RACHS-1 was associated generally with higher mortality rate. This is in keeping with evidence from other studies that have identified a positive correlation between RACHS and mortality rates (57).

The economic impact was not well covered across the studies with only 6 studies discussing this. Further the cost estimates varied substantially across programmes, reflecting differences in healthcare settings, costing methodologies, resources, and reporting years. Only two studies looked at DALYs, but it was clear with each that these programmes made huge improvements in DALYs of the participants. Economic outcomes such as improvement in DALYs or QALYs have their limitations but they are important and difficult to ignore to policy makers and government hence there is a need for studies to display the economic impact of their programmes (58).

Capacity building initiatives are integral to improving sustainability of programmes (54) and most of the studies described at least one capacity building initiative. Our findings show that more than half of the programmes included training and education and systems strengthening approaches. However, fewer studies focussed on infrastructure development. Infrastructure development can take time and effort and therefore may not be popular amongst programmes. Successful programmes require a multi-faceted and integrated approach (54).

### 4.1 Strengths and limitations

This review has several strengths. It maps many studies from diverse developing economies. The additional depth and focus on programme characteristics and sustainability of paediatric heart disease programmes, makes our review unique. Sustainability is a crucial topic of interest in a world with increasingly limited resources. Our review provides a comprehensive view of the various programmes and their outcomes. This can help policy makers and health leaders design efficient paediatric heart disease treatment programmes that deliver their purpose.

However, there were also some limitations in our review. Although measures with taken to reduce selection bias, the absence of duplicate screening throughout all stages remains a limitation and may have introduced selection bias. A risk of bias assessment was not carried out in keeping with methodology guidance for scoping reviews. The exclusion of any unpublished data means we might have missed other similar programmes. We only included collaborative programmes; however, it was beyond the scope of this review to assess the specific nature or extent of these collaborations.

This data was collected from diverse healthcare settings and included heterogeneous data which poses challenges in drawing uniform conclusions. Mortality was well reported across studies, but due to heterogeneity in mortality definitions, programme characteristics and case complexities the outcomes could not be pooled and instead were summarised descriptively. Economic impact, was poorly reported, highlighting the need for future studies to address these gaps. Lastly, there was a lack of long-term follow-up in many of the studies and this limits the ability to assess the longer term sustained impact of these programmes on health outcomes, economic benefits, and capacity building.

### 4.2 Policy implications

Our review suggests the collaborative paediatric heart disease treatment programmes help in addressing the gap to access of paediatric cardiac services. The findings of our review can support further initiatives for paediatric heart disease treatment programmes and can help health leaders in designing future programmes and in allocation of resources.

Our review also showed that there is a critical gap in evidence of economic impact and focussed research is necessary to quantify the economic outcomes, which is essential for understanding the broader financial implications of collaborative programmes. Many programmes like these are inherently built to address a strong clinical need rather than as a research initiative and therefore do not have standardised mechanisms for prospective data collection and evaluation which then limits sharing of expertise and research. We believe establishing standardised reporting frameworks or registries for paediatric cardiac global health programmes could rapidly enhance shared learning and allow consistency in data collection, and strengthen future evaluation of clinical outcomes, capacity building, and sustainability.

Finally, we would recommend programmes to introduce policies which prioritise building local capacity through training and education, building local infrastructure and systems strengthening approaches. The aim should be for a local unit to be independent and able to provide continuous service. Funding models should not be over-reliant on a single source of funds but rather work as 2P or 3P partnership models as this distributes risk and improves sustainability. Overall, a holistic approach is needed to provide sustainable and effective care in resource-limited settings.

## 5 Conclusion

Our review maps diverse collaborative paediatric heart disease treatment programmes and provides insight into their key characteristics, approaches and reported outcomes. We show that these programmes in developing countries can help improve access to paediatric cardiac care in resource limited settings. Moreover, the included programmes, describe favourable clinical outcomes although heterogeneity between studies limited comparison of these health outcomes. The studies describe efforts to integrate capacity building initiatives which can improve the potential to be more equitable and sustainable over time. Our analysis of these studies suggests that long term programmes can facilitate with capacity building better than short term “safari” missions. However, collaborative programmes face various challenges, and sustained support from stakeholders including governments as well as NGOs can help strengthen their impact.

There is a clear need for further research to evaluate the cost-effectiveness, long term effectiveness and sustainability of these programmes to better understand how collaborative models can be optimised to support equitable and sustainable paediatric cardiac care globally. Ultimately, a systems approach is required to tackle the global disparities in paediatric heart disease and bridge the gap to improving paediatric cardiac care globally.

## Data Availability

The original contributions presented in the study are included in the article/Supplementary Material, further inquiries can be directed to the corresponding author.

## Glossary

Acquired Heart Disease: Heart disease that develop after birth due to factors such as lifestyle, infection, or aging.
Congenital Heart Disease: Heart abnormalities that are present from birth.
Disability Adjusted Life Years: A measure of overall disease burden, representing the total number of years gained due to illness, disability, or premature death.
Intensive Care Unit Stay (ICU): A specialised unit in a hospital providing intensive treatment and monitoring for critically ill patients.
Length of Stay (LOS): The duration of a patient's stay in a hospital or healthcare facility, often measured in days.
Paediatric Heart Disease: Umbrella terms comprising both congenital and acquired heart disease.
Quality Adjusted Life Years: A measure that integrates both the length and quality of life to evaluate the effectiveness of medical treatments.
Risk Adjustment for Congenital Heart Surgery (RACHS-1): A standardised classification system that categorises the complexity of congenital heart surgeries. The categories range from 1 to 6, with 6 being the most complex category.
Standardised Mortality Ratio (SMR): A ratio that compares the observed number of deaths in a study group to the expected number of deaths in the standard population.
