# Supplementary material for: A scoping review exploring the health impact and sustainability of collaborative paediatric heart disease treatment programmes in developing countries
Source: Front Pediatr. 2026 Aug 11;14:1921366. doi: 10.3389/fped.2026.1921366 (PMC13504811; doi:10.3389/fped.2026.1921366)
Supplement: Supplementary file 1 [file Table1.docx]

Supplementary Material

# 1 Supplementary Data

# Supplementary Table 1: Health outcomes table

| Study ID | Mortality | Mortality definition | Mortality reduction (%) | Complications (Short / Long term) | LOS (days) | ICU stay  (days) |
| --- | --- | --- | --- | --- | --- | --- |
| Ali & Medani (2022) | - | - | - | - | - | - |
| Balachandran et al. (2015) | 3.10% | In-hospital | 2.10% | **Infection**: 11.9% bacterial sepsis; 6.4% surgical site infection | - | 4.2 (median) |
| Bawiskar et al. (2020) | - | - | - | - | - | - |
| Cardarelli et al. (2018) | 8% | Overall | - | - | - | - |
| Cohen et al. (2001) | 5.2% | In-hospital | - | - | - | - |
| Croti et al. (2019) | 12.4% | 30 day | 7.00% | **Infection**: 10% bacterial sepsis; 3.3% surgical site infection; 13% Other infections | - | - |
| Das et al. (2024) | 5.20% | In-hospital | - | - | - | - |
| Rassi et al. (2020) | 2.80% | Overall | - | **Infection**: 12.4%; Pulmonary: Pneumonia; **Re-operation**: 14%  **Other**: Peritoneal dialysis 5.3%; Acute neurologic complications inc seizures 2.1% | 12.2 (mean) | 5.7 days (mean) |
| Furniss et al. (2023) | 2.9% | 5 – year follow up | - | 13.5% (12 patients) experienced complications; **Pulmonary**: diaphragmatic paralysis, chylothorax, pulmonary oedema; **Cardiac**: Post pericardiotomy syndrome, cardiac tamponade, arrhythmia; **Other**: short-term renal failure, laryngeal nerve paralysis; **Long** term: 1 patient had third AV block needing pacemaker. | 12 days (median) | 2 days (median) |
| Henry et al. (2005) | 3.40% | Overall | - | **Infection**: Superficial wound infection (not mentioned number) **Bleeding**: Most common complication. **Cardiac**: Pericardial effusions, tamponade (0.5%), 1.4% had complete AV block needing external pacing. **Re-operation**: 2.9% returned to theatre due to bleeding; 0.5% (1 patient) had aortic valve damage needing St Jude prosthetic valve.; **Long term**: 0.5% needed pacemaker (1 patient). 0.5% had neurological weakness (1 patient) | - | - |
| Ho et al. (2011) | 5.40% | Overall | - | **Infection**: 2.8% infective endocarditis  **Other**: 2.8% haemolysis post PDA; 11% residual defects | - | - |
| Isaac et al. (2017) | 5% | Overall | 4% | - | - | - |
| Kim et al. (2023) | 2.73% | Overall | 8.00% | - | - | - |
| Lacour-gayet et al. (2022) | 5.50% | Peri-op | - | - | 9 days (mean) | - |
| Larrazabal et al. (2007) | 10% | Overall | - | Complication was present in 341 of 1215 procedures (28%); **Infection**: In 13.8% (n=168); **Bleeding**: Bleeding at 2% (n=25), capillary leak syndrome at 2% (n=24); **Pulmonary**: pleural effusion at 8% (n=92); pneumothorax at 3% (n=34). **Cardiac**: Low cardiac output at 2% (n=27), and arrhythmia at 2% (n=21). | 6 days (median) | 1 day (median) |
| Leon-Wyss et al. (2009) | 8.30% | Overall | 2.00% | Complications occurred in 523 of the 2,630 procedures (20%).; **Infection**: Present in 17% (n=447) of the cases.; **Bleeding**: bleeding (2%; n=52); capillary leak syndrome (2%; n=52); **Pulmonary**: Pleural effusion (8%; n=210), pneumothorax (2%; n=52); **Cardiac**: Low cardiac output (4%; n=105); arrhythmia (2%; n=52). | 6 days (median) | 3 days (median) |
| Liu (2009) | 3% | Overall | 7% | - | - | - |
| Marianeschi et al. (2021) | 0.80% | Post-op | - | - | - | - |
| Marianeschi et al. (2022) | 2% | Overall | - | - | 5 days (mean) | 3 days (mean) |
| Nair et al. (2021) | 2.40% | 30-day | 41.00% | - | - | - |
| Novick et al. (2005) | 9.50% | Overall | 8.70% | - | - | - |
| Nunes et al. (2017) | 4.20% | 30-day | - | **Infection**: 3.6%; **Pulmonary**: Pericardial/pleural effusion 4.4%; **Cardiac**: Post-op arrhythmia 2.9%; **Re-operation**: 4.3% re-operation rate; **Long term:** Pacemaker 1.8% | 9 days (median) | 2.3 days (median) |
| Park et al. (2022) | - | - | - | - | - | - |
| PirosKulandasamy Pillai et al. (2016) | 1.70% | Post-op | - | - | - | - |
| Sasson & Schachner (2021) | - | - | - | - | - | - |
| Shidhika et al. (2019) | 3.80% | 30-day | - | 156 patients, 80 (51.3%) had postoperative complications. **Infection** 1.2%; **Bleeding**: Bleeding 1.3%; **Pulmonary**: Pneumonia 26%; PH crisis 5.8%; Persistent pleural drainage 3.9%; Pulm reperfusion injury 1.3%; Diaphragm paralysis 1.3%; Chylothorax 0.6%; **Cardiac**: Arrhythmia 5.8%; Pericardiotomy syndrome 2.6%; Myocardial ischaemia 0.6%; **Other**: Systemic to pulm shunt thrombosis 0.6% | - | 5 days (median) |
| Song et al. (2023) | 0.10% | Mortality at 1 year follow up | - | - | - | - |
| Tefera et al. (2015) | 1% | In-hospital |  | **Re-operation**: 1.7% device embolisation requiring surgical retrieval; 1% mitral valve replacement due to severe MR following balloon dilatation, 0.4% stroke.  **Long term:** 0.4% neurological complication (embolic stroke leading to hemiparesis) | - | - |
| Tefuarani et al. (2002) | 6% | Post-op | - | 40% had post-op complications; **Pulmonary**: 38% pulmonary; **Cardiac**: 51% were cardiac complications, and **Other**: 7% were other complications | - | - |
| Tefuarani et al. (2007) | 1.90% | In-hospital | - | **Re-operation:** 1.9% re-operations | - | - |
| Tomita et al. (2013) | - | - | - | - | - | - |
| Wallen et al. (2018) | 8.20% | In-hospital | 5.80% | **Bleeding**; **Re-operation**: 11.1%; **Other**: Re-intubation 8.1%.; **Long term:** 0.5% pacemaker insertion | - | - |
| Youssef et al. (2024) | 4.10% | In-hospital | - | - | 12.2 days (mean) | 5.7 days (mean) |
| Zain et al. (2019) | 11% | In-hospital | - | - | - | - |
| Zhang et al. (2021) | 0% | Overall | - | **Pulmonary**: ARDS 15.9%; **Re-operation**: Re-exploration 0.4%; **Other:** Acute liver injury 1.3%; Delayed wound healing 1.3%; AKI 1.3%; ICH 0.4%; Lower limb ischaemia 0.4%  **Long term:** Epilepsy 0.4% | - | - |
